# The effects of molecular crowding and CpG hypermethylation on DNA G-quadruplexes formed by the *C9orf72* nucleotide repeat expansion

**DOI:** 10.1038/s41598-021-02041-4

**Published:** 2021-12-01

**Authors:** Kadir. A. Ozcan, Layla T. Ghaffari, Aaron R. Haeusler

**Affiliations:** 1 Department of Neuroscience, Jefferson Weinberg ALS Center, Vickie and Jack Farber Institute for Neuroscience, Thomas Jefferson University, Philadelphia, PA USA; 2 Present Address: Department of Neuroscience, Jefferson Weinberg ALS Center, Vickie and Jack Farber Institute for Neuroscience, Thomas Jefferson University, 900 Walnut Street, JHN suite 410, Philadelphia, PA 19107 USA

**Keywords:** DNA, Molecular modelling, Dementia, Motor neuron disease, Neurodegeneration, Neurodegenerative diseases, Neuromuscular disease, Spinal cord diseases, Molecular conformation

## Abstract

A nucleotide repeat expansion (NRE), (G_4_C_2_)_*n*_, located in a classically noncoding region of *C9orf72* (C9), is the most common genetic mutation associated with ALS/FTD. There is increasing evidence that nucleic acid structures formed by the C9-NRE may both contribute to ALS/FTD, and serve as therapeutic targets, but there is limited characterization of these nucleic acid structures under physiologically and disease relevant conditions. Here we show in vitro that the C9-NRE DNA can form both parallel and antiparallel DNA G-quadruplex (GQ) topological structures and that the structural preference of these DNA GQs can be dependent on the molecular crowding conditions. Additionally, 5-methylcytosine DNA hypermethylation, which is observed in the C9-NRE locus in some patients, has minimal effects on GQ topological preferences. Finally, molecular dynamic simulations of methylated and nonmethylated GQ structures support in vitro data showing that DNA GQ structures formed by the C9-NRE DNA are stable, with structural fluctuations limited to the cytosine-containing loop regions. These findings provide new insight into the structural polymorphic preferences and stability of DNA GQs formed by the C9-NRE in both the methylated and nonmethylated states, as well as reveal important features to guide the development of upstream therapeutic approaches to potentially attenuate C9*-*NRE-linked diseases.

## Introduction

The *C9orf72* (C9) nucleotide repeat expansion (NRE) mutation, consisting of a repeated hexanucleotide (G_4_C_2_)_*n*_ located in a classically noncoding region of the gene, is the most prevalent genetic mutation associated with the neurodegenerative diseases amyotrophic lateral sclerosis (ALS) and frontotemporal dementia (FTD)^1,2^. ALS and FTD are two progressive and fatal neurodegenerative conditions that are ultimately caused by the death of motor and cortical neurons, respectively^3,4^. Typically, most individuals carry < 24 (G_4_C_2_) repeats, but symptomatic ALS/FTD patients often carry > hundreds or thousands of repeats^5,6^. It has been proposed that these age-related diseases linked to the C9-NRE mutation may be caused by three non-mutually exclusive pathogenic mechanisms: (1) reduced transcription of the NRE region results in C9ORF72 haploinsufficiency^2,7–9^; (2) repeat containing-RNA produced from the NRE sequesters or alters dynamics of essential nucleoproteins^7,10^; and/or (3) the non-AUG-dependent translation of the bidirectionally transcribed repeats to produce five unique dipeptide repeats (DPRs) that can confer cellular toxicity^10,11^. Extensive work in C9-NRE patient tissue and disease models has identified numerous overlapping and divergent cellular defects and pathological features potentially associated with these three putative C9-NRE-linked pathogenic mechanisms for ALS/FTD^12–14^. However, despite the cumulative advancements in our understanding of C9-NRE-linked disease mechanisms over the past decade there are currently limited therapies to prevent the pathogenic cascade^15^.

Increasing studies show that pharmacologically targeting nucleic acids may be a tractable therapeutic approach to treat human diseases, including neurodegenerative disorders and cancer^16–22^. For example, recent pharmacological approaches employing small molecules to target unique DNA hairpin structures in a (CAG)_*n*_ NRE mutation associated with Huntington’s disease can induce DNA repeat contractions, thereby reducing disease severity, which is correlated with repeat-length^23^. Additionally, small molecules targeting DNA G-quadruplex (GQ) structures formed in repetitive telomeric regions or in oncogenic regions have demonstrated anti-cancer properties, and these GQ structures are increasingly appreciated as potential therapeutic targets for neurodegenerative diseases^20,22,24–26^. Therefore, molecular therapies directed at nucleic acid structures formed in the C9-NRE mutation DNA region are a promising therapeutic target for mitigating pathological hallmarks of C9-NRE-linked ALS/FTD disease in vitro and in vivo.

**Figure 1:**
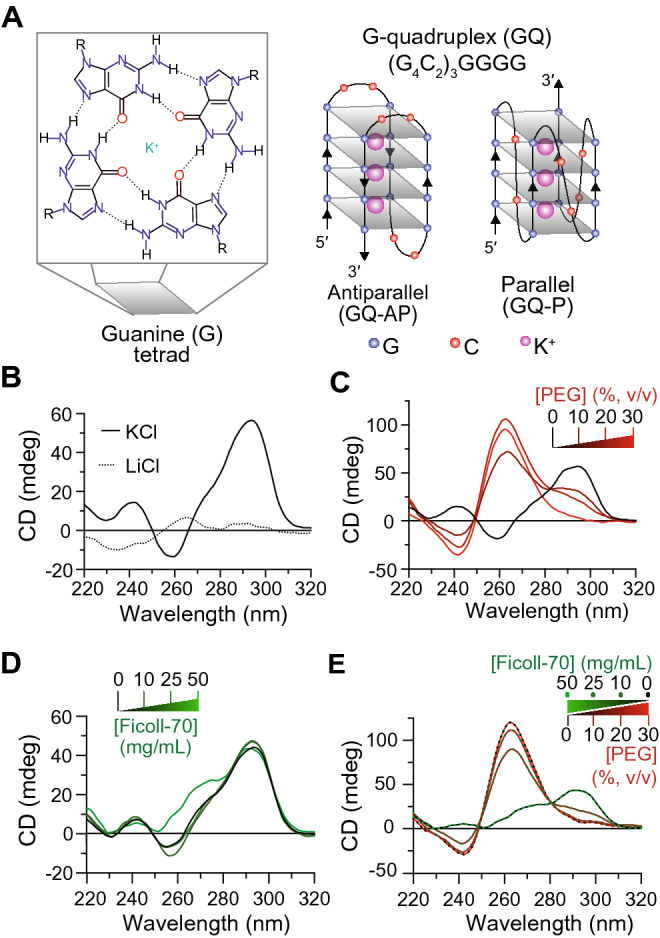
Molecular crowding conditions can impact GQ structural preferences of the *C9-NRE* DNA. **(A)** Schematic representation of the guanine tetrad that stack to form an anti-parallel (left) and/or parallel (right) DNA G-quadruplex topologies adopted by the C9-NRE. Gray planes depict the Guanine tetrads. Blue, red, and purple spheres represent guanine residues, cytosine residues, and potassium ions, respectively. (**B**) Representative CD spectra of *C9-*22mer oligo, 10 µM, in the presence of 100 mM KCl (solid line) or 100 mM LiCl (dotted line) at 25 °C demonstrate that K^+^ promotes the formation of GQ-AP. (**C**) Representative CD spectrum for the *C9*-22mer oligo in the presence of 100 mM KCl and increasing molecular crowding agent of 0, 10, 20, and 30% [PEG-200] (v/v) are denoted by the black to red line transition. (**D**) CD spectrum for the *C9*-22mer in the presence of increasing molecular crowding agent 0, 10, 25, and 50 [Ficoll-70] (mg/mL) are denoted by the black to green line transition. (**E**) Representative CD spectrum for decreasing [Ficoll-70] combined with increasing [PEG-200] are shown for the *C9*-22mer oligo.

The expanded C9-NRE DNA is structurally polymorphic and forms non-canonical structures including GQs^7,27–30^, but it is still unknown if these structures could be an effective upstream therapeutic target for preventing C9-NRE-linked ALS/FTD. GQs are non-canonical nucleic acid structures that are composed of four guanine residues that base pair through Hoogsteen hydrogen bonds to from a planar tetrad^31^. These tetrads can stack together to form helical structures stabilized by π-π interactions and by specific monovalent cations between tetrads (depicted in Fig. 1A). Structural insights provided by biophysical studies examining varying lengths of the C9-NRE DNA sequence has demonstrated that this repetitive region can form several stable intramolecular and/or intermolecular anti-parallel GQ (GQ-AP) or parallel GQ (GQ-P) topological configurations^7,27,28^, with the topology defined by the relative orientations of the phosphate backbone in the GQ structures^31^ (Fig. 1A). NMR spectroscopy and X-ray crystallography studies have generated detailed atomic resolution structures for some of these possible C9-NRE GQ-AP and GQ-P structural conformations^29,30^. Importantly, these atomic structures could be utilized to screen and identify therapeutic candidates that might modify the stability of these structures and thus alter the C9-NRE-linked ALS/FTD disease cascade in patients. However, it is currently unknown if more physiologically relevant cellular conditions or disease-relevant DNA modifications, such as DNA hypermethylation identified in some patient cohorts^32–34^, lead to different GQ topological or structural preferences. Furthermore, the dynamics and structural ensembles for these different unimolecular C9-NRE DNA GQ structures have not been fully examined^35^. Therefore, determining the structural preferences and structural ensembles under more physiologically relevant conditions is crucial to understand if targeting nucleic acid structures formed by C9-NRE DNA can be an efficacious treatment strategy for C9-NRE-linked ALS/FTD.

In this work, we show that C9-NRE DNA GQ structures can transition from a GQ-AP topological preference to a GQ-P topology under certain molecular crowding conditions that attempt to recapitulate molecular crowding conditions encountered within the nuclear environment in cells or potentially membraneless organelles^36–38^. Additionally, 5-methylcytosine (5mC) hypermethylation of the C9-NRE DNA sequence, which mimics DNA cytosine-phospho-guanine (CpG) hypermethylation identified in some patient cohorts carrying the C9-NRE mutation^34^, has minimal effect on GQ structural preferences under different molecular crowding conditions or on the overall stability in thermal melt assays. Molecular dynamics simulations on unimolecular methylated or nonmethylated GQ DNA structures supports these in vitro results showing that C9-NRE GQ structures are stable, and that methylation status has minimal effect on the overall stability or structural ensembles in these simulations. Moreover, most structural fluctuations in these simulations are largely limited to the cytosine-containing loop regions, and these results indicated that the dynamic loop regions may provide unique opportunities for pharmacological specificity. Together, these findings further expand our knowledge of the structural preferences of DNA GQs formed within the C9-NRE mutation locus under more physiologically relevant conditions and provides new, upstream molecular structures that may serve as therapeutic targets to prevent the C9-NRE-linked neurodegenerative disease cascade.

## Results

### Molecular crowding conditions can affect the topological distributions of G-quadruplexes formed by the C9-NRE DNA

The disease associated C9*-*NRE DNA mutation has been previously shown to be structurally polymorphic; varying lengths of DNA oligos derived from the NRE region can form canonical Watson:Crick double-helix bDNA as well as non-canonical hairpins and GQs with the diversity of these structures possibly increasing with repeat length^7,27–30^. However, the impact of the cellular environment on these different DNA structures is unknown. It has been shown that GQ topological preferences are influenced by molecular crowding conditions. For example, a parallel GQ often forms under conditions of osmotic stress^39^, while other molecular crowding reagents can support either GQ-P or GQ-AP structures for certain DNA sequences^40^. Therefore, we first set out to topologically identify possible unimolecular GQ-AP and GQ-P structures (Fig. 1A), by employing circular dichroism (CD) spectroscopy on an oligonucleotide from the coding strand of the C9-NRE mutation, (G_4_C_2_)_3_GGGG (*C9-*22mer) (Supplementary Table S1). CD spectroscopy experiments were performed using standard spectroscopy conditions in the presence of well-established monovalent cations, potassium (K^+^) or lithium (LI^+^) that either do or do not stabilize GQ-structures, respectively^31^. The results from these spectroscopy studies show that in the presence of monovalent K^+^, the *C9-*22mer display a strong positive wavelength peak at 295 nm and a negative peak at 260 nm (Fig. 1B)^7^, which are characteristic CD spectrum signatures of a GQ-AP topology. In the presence of monovalent Li^+^, which there is a very slight positive wavelength peaks at 260 nm and 290 nm, and a negative peak at 230 nm (Fig. 1B), which indicates the *C9-*22mer does not form a GQ structure in the presence of Li^+^ but may instead be forming a hairpin structure^7^. The stabilization of GQ structures in the presence of K^+^ and not in the presence of Li^+^ are also recapitulated with a longer coding strand C9-NRE length, consisting of the DNA oligo (G_4_C_2_)_8_ (*C9*-48mer) (Supplementary Table S1 and Fig. S1A). These findings for K^+^-dependent stabilization of GQ-P and/or GQ-AP structures have been shown with other GQ-forming DNA sequences^31^, and directly shown using C9-NRE oligos ranging up to 10 (G_4_C_2_) repeats in length or indirectly shown in a plasmid containing pathogenic C9-NRE lengths^7^.

We then set out to determine if molecular crowding conditions analogous to those that are found in the nucleus of the cell may influence the DNA topological structural preferences. Molecular crowding agents have been shown to influence GQ-AP and GQ-P topological distributions, with the physical characteristics of the crowding environment being critical in these studies^36,37^. To investigate these potential effects, we performed CD spectroscopy on the *C9*-22mer in the presence of increasing concentrations of the widely utilized molecular crowding agent polyethylene glycol 200 (PEG) and/or Ficoll-70 (Ficoll). The CD spectroscopy results show that by increasing PEG concentrations to mimic environments with increased dehydration or osmotic stress^39,41^, the topological distribution of GQs formed by C9-NRE DNA transitions from a GQ-AP to a GQ-P topological preference, which is evident by the respective PEG concentration-dependent decreasing amplitude of the GQ-AP 295 nm peak and concomitant increase in the intensity of the corresponding GQ-P 260 nm peak (Fig. 1C)^7,31^. Even at relatively low PEG concentrations (10%) there is a substantial redistribution from GQ-AP to a predominantly GQ-P topology with a positive 260 nm peak and a negative 240 nm peak, and minor population contributions of GQ-AP as shown in the CD spectrum and the spectral decomposition fractional component analyses (Fig. 1C and Supplementary Fig. S2A). However, in the presence of the molecular crowding agent Ficoll, which has been indicated as a better mimic of the cellular nuclear environment for modeling telomeric GQ structures in contrast to PEG^40,41^, the *C9*-22mer maintains the GQ-AP topological distribution as shown by the stable CD spectra profiles for GQ-AP upon increasing concentrations of Ficoll (Fig. 1D and Supplementary Fig. S2B). We then examined the combination of different Ficoll and/or PEG concentrations, and we found that the dehydrating environment induced by PEG dominates the topological preferences—a PEG-dependent transition from a GQ-AP to a GQ-P topology is observed for the CD spectra independent of the Ficoll concentration (Fig. 1E and Supplementary Fig. S2C). Finally, we expanded these *C9*-22mer studies to the longer repeat length oligo, a *C9*-*48*mer, and we observed almost identical CD spectra results for all molecular crowding conditions as seen with the *C9*-22mer (Supplementary Fig. S1). Overall, these molecular crowding mimetic results are consistent with previous studies that show PEG induces changes in telomeric DNA GQ structure in, and these changes are not observed in the presence of molecular crowding mimetics such as Ficoll or crude intracellular extracts^40,41^. However, these results indicate that the C9-NRE DNA can form both GQ-AP and GQ-P topologies, with these topological preferences influenced by the molecular crowding microenvironmental conditions of the cell.

### The structure and stability of *C9orf72* repeat locus DNA GQs are largely unaffected by CpG hypermethylation

**Figure 2:**
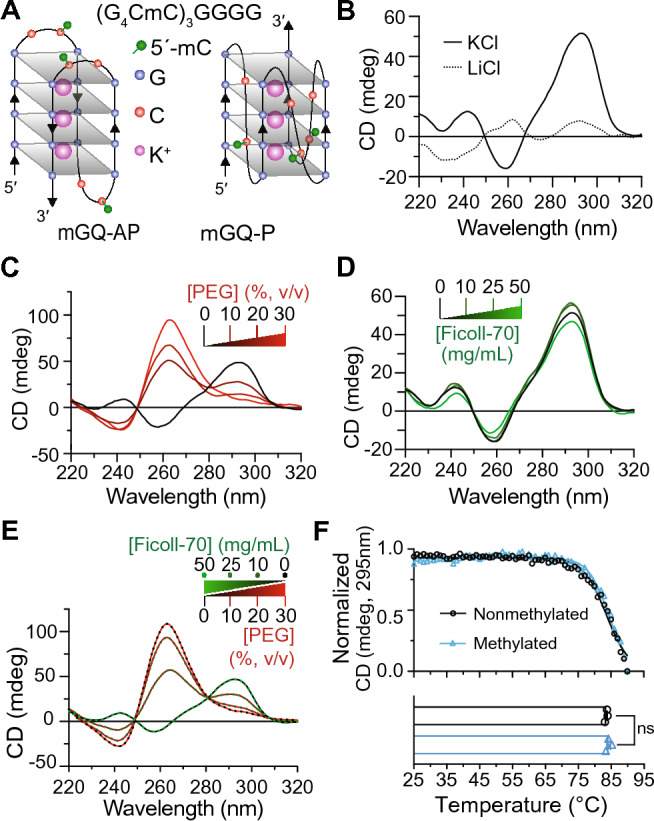
CpG hypermethylation of C9-NRE DNA has minimal effects on GQ topological distributions or stability. (**A**) Representation of methylated antiparallel (mGQ-AP) and parallel (mGQ-P) GQ examined using a methylated *C9*-22mer (m*C9*-22mer). The green spheres denote the three locations of 5-methylcytosine (5mC) CpG modifications on the mC9-22mer. (**B**) Representative plot for CD spectra for 10 µM of the m*C9*-22mer in the presence of 100 mM KCl (solid line) or 100 mM LiCl (dotted line) at 25 °C. (**C**) Representative plots are shown for CD spectra for 10 µM of the m*C9-*22mer in the presence of 100 mM KCl and increasing molecular crowding agent of 0, 10, and 20% [PEG-200] (v/v) are depicted by the black to red line transition. (**D**) CD spectrum for the mC9-2mer in the presence of increasing molecular crowding agent 0, 10, 25, and 50 [Ficoll-70] (mg/mL) are denoted by the black to green line transition. (**E**) Representative CD spectra for the m*C9*-22mer are shown in the presence of decreasing [Ficoll-70] combined with increasing [PEG-200]. (**F**) Melt comparison of 10 µM nonmethylated (*C9*-22mer, black line and empty circles) and methylated (m*C9*-22mer; cyan line and triangles) *C9*-22mer in the presence of 100 mM KCl are plotted. Circles denote the average value with lines showing the sigmoidal fit to the averaged data. The 295 nm wavelength was measured at a temperature range of 25–90 °C. The bar graph below shows the calculated melting temperature (T_m_) obtained from the sigmoidal fits as the mean ± SEM for *n* = 3 with a non-significant (NS) *p*-value = 0.3.

DNA bisulfite sequencing and methylation sensitivity assays performed on ALS patients carrying the C9-NRE mutation have shown that the *C9orf72* promoter and NRE region itself can have 5mC hypermethylation at CpG sites^32–34^. The extent of CpG methylation for the repeat itself is unknown and may vary among patient cohorts^34,42^. However, CpG hypermethylation at the C9-NRE locus has been shown to have an inverse relationship between methylation levels and C9-NRE-linked pathological features, and thus, has been proposed to modify disease onset and progression^32,33^. Therefore, to further determine if the presence of 5mC hypermethylation may alter C9-NRE DNA structural distributions in vitro, we performed CD spectroscopy on the *C9-*22mer (described earlier) with the addition of 5mC modifications at all three available CpG sites (m*C9-*22mer) (Fig. 2A and Supplementary Table S1). The CD spectra for the m*C9-*22mer (Fig. 2B) show that in the presence of K^+^, the methylated C9 oligo maintains a GQ-AP topology, indicated by the canonical positive wavelength peak at 295 nm and slight negative peak at 260 nm. This pattern is not observed when the GQ-stabilizing K^+^ was substituted with Li^+^. These results for the m*C9*-22mer are consistent with previous GQ-AP topological CD spectroscopy signatures and cation-specific GQ stabilization.

We then examined if specific molecular crowding environment conditions can alter the GQ-AP and GQ-P topological distributions for the m*C9*-22mer. Consistent with the findings for the nonmethylated *C9-*22mer (Fig. 1C), the methylated m*C9-2*2mer displays CD spectra profiles that show a PEG concentration-dependent transition from a GQ-AP to a GQ-P topological preference (Fig. 2C and Supplementary Fig. S3A). In the presence of only Ficoll, the m*C9-22*mer maintains the GQ-AP topological CD spectra signature (Fig. 2D and Supplementary Fig. S3B), but when PEG is titrated in substitution of Ficoll, the CD spectra show a clear PEG concentration-dependent transition from the GQ-AP to the GQ-P topology (Fig. 2E and Supplementary Fig. S3C) as observed with the nonmethylated *C9*-22mer (Fig. 1 and Supplementary Fig. S2). Finally, we then examined if PEG or Ficoll had similar effects on longer hypermethylated repeat lengths. Employing a CpG hypermethylated *C9*-48mer (*mC9*-48mer), we show that the results for the shorter repeat length m*C9*-22mer in the presence of different cations or molecular crowding conditions also extend to the longer repeat length m*C9*-48mer (Supplementary Fig. S4). Together, these results indicate the hyper- or hypo-methylation of the C9-NRE DNA has minimal effects on the GQ topological distributions for the C9-NRE DNA.

To investigate the effects of 5mC methylation on GQ-AP stability in vitro, we performed thermal stability assays on the nonmethylated and methylated *C9-*22mers. The results from these experiments (Fig. 2F) demonstrate that methylation does not have significant effects on the stability of the DNA GQ-AP, with melting temperatures calculated to be 84.3 °C and 83.6 °C for the methylated and nonmethylated *C9-*22mers, respectively. These results are consistent with previous findings that showed CpG methylation on similar C9-NRE GQ-forming oligos has minor effects on the structural stability in CD spectroscopy thermal melt assays^22^. In summary, the CD spectroscopy and thermal stability assay results indicate that the methylation status of the C9-NRE itself has minimal effects on the formation and stability of GQ-AP or GQ-P structures formed within this region.

### DNA G-quadruplexes formed by the C9-NRE are stable in molecular dynamics simulations

**Figure 3:**
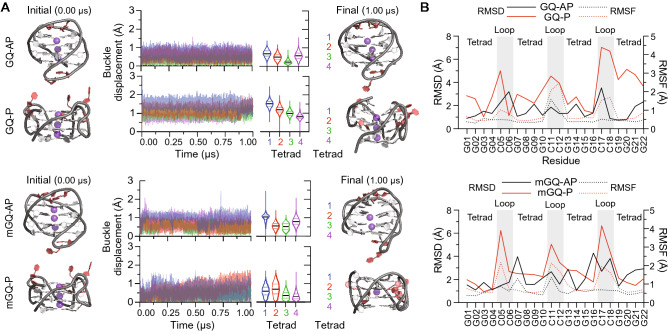
Nonmethylated and methylated DNA GQ structures formed by the *C9-NRE* are stable in MD simulations. (**A**) The buckle displacement values for each tetrad of the nonmethylated (top; GQ-AP, GQ-P) and methylated (bottom; mGQ-AP, mGQ-P) GQ are plotted over the 1 µs MD simulation time course. The frequency distribution of buckle displacement data, located to the right of the plotted buckle displacement time course, are shown as violin plots with solid lines denoting the mean and dotted lines the quartiles. The respective initial (0 µs) and final (1 µs) MD simulation representative GQ atomic structures are shown on the left and right of the buckle displacements graphs, respectively. (**B**) The mean RMSD (left *y*-axis, solid lines) and RMSF (right *y*-axis, dashed lines) analyses are plotted per residue for nonmethylated (top; GQ-AP, GQ-P) and methylated (bottom; mGQ-AP, mGQ-P). Individual residues are labeled from 5′ to 3′ below the *x*-axis with ‘G’ and ‘C’ representing guanine and cytosine residues, respectively. Residues that are part of tetrads versus loops (gray strips) are designated at the top of the plots.

We then examined the stability and dynamics of the DNA GQ structures formed by the C9-NRE. Our results (Figs. 1 and 2) and previous findings have shown that the (G_4_C_2_)_*n*_ DNA can adopt GQ-AP topology based on CD spectroscopy, DMS protection assays, GQ nanobody-specific immunoprecipitations, and NMR/X-ray structural studies^7,27,30,43^, and it has been demonstrated that these GQ-AP topological arrangements are thermodynamically stable *in vitro*^7,27^. However, molecular dynamics (MD) simulations to evaluate the stability of these unimolecular structures and potential conformational ensembles have not been performed. Therefore, to provide insight into the structural stability and dynamics of the GQs, we performed MD simulations over a 1 µs duration on C9-NRE GQ structures that we generated from existing and/or through modifications of reported NMR structures (see Materials and Methods). The buckle displacement analyses from these MD simulations, which provides an overall measurement of GQ structural stability, demonstrates that all tetrads for both GQ-AP and GQ-P have limited structural deviations over the entire 1 µs simulation (Fig. 3 and Supplementary Table S2). Moreover, the overall root mean square deviation (RMSD) indicates the C9-NRE GQ structures have limited variation over the simulation time course with the cytosine-containing loop regions contributing largely to the overall RMSD values (Fig. 3B, Supplementary Table S2, and Supplementary Fig. S5A). The root mean square fluctuation (RMSF), which measures the dynamics of residues over the entire course of the simulation relative to the average RMSD, further demonstrates that the guanine residues in the tetrads have significantly lower structural variability compared to the cytosine-containing loop residues (Fig. 3B, Supplementary Table S2, and Supplementary Figs. S5A and S6). Overall, these MD simulation results indicate that the dynamics and stabilities of the GQ-AP and GQ-P formed by the C9-NRE DNA are similar, and the four tetrads are highly stabilizing factors for these unique nucleotide structures.

We then performed MD simulations on 5mC methylated GQ-AP (mGQ-AP) and GQ-P (mGQ-P). The previous GQ structures used in MD simulations described earlier were modified to contain three CpG 5-methylcytosine modifications (Fig. 3A) and were then subjected to 1 µs MD simulations. The results of the MD simulations for mGQ-AP and mGQ-P (Fig. 3, Supplementary Table S2, and Supplementary Fig. S5B) show minimal changes in buckle displacement over the course of the simulations. Both the RMSD and RMSF values for both methylated GQs also indicate that the 5mC modifications have little effect on GQ structure stability and that the cytosine-containing loop regions are more motile than the tetrads and are the primary contributors to the overall structural deviations for the methylated C9-NRE DNA GQs over time (Fig. 3B, Supplementary Figs. S5 and S6, and Supplementary Table S2). In conclusion, the GQ topological configurations are stable in MD simulations and methylated GQs exhibit almost equivalent molecular dynamics to the nonmethylated C9-NRE DNA GQ structures.

## Discussion

There is increasing evidence that GQs contribute to normal biology and disease through several unique mechanisms within the central dogma of molecular biology^31,44^. Specifically, at the DNA level, GQ-forming regions: have been identified throughout the human genome^45,46^, have been shown to be crucial regulators of the transcriptomic and the epigenomic landscape^47–49^, and are highly prevalent in mouse brain tissue^50^. Therefore, the potential role of non-canonical DNA structures in modifying age-related neurodegenerative processes are of increasing interest^12,51,52^, and therapeutic targeting of GQ structures to treat age-related diseases is being actively pursued^24–26^. Here we explored the DNA GQ structural landscape of the ALS/FTD-linked C9-NRE mutation under different physiological conditions in vitro*,* and the dynamics of these structures in silico. The results of this work demonstrate that the human C9-NRE can form stable DNA GQ-AP structures, and under specific dehydrated molecular crowding conditions similar to environments or microenvironments undergoing osmotic stress, the DNA GQ-P is the dominant structure. Direct CpG hypermethylation of the C9-NRE, which is observed in some patient cohorts^32–34^, has minimal effects on the overall stability or topological preferences. Finally, our molecular dynamic simulations of the C9-NRE GQs supports that these structures are highly stable with the cytosine-containing loop regions contributing the most to the overall structural dynamics and deviations over time. Together, these studies provide a structural platform for the development of pharmacological tools targeting GQ structures in efforts to treat C9-NRE-linked diseases.

Numerous neurodegenerative-associated proteins have been linked to aberrant liquid–liquid phase separation (LLPS) *in vitro*^53^, and DNA GQs have also been shown to participate in the LLPS process^54^. Additionally, the C9-NRE-containing RNA has been shown to have increasing LLPS properties with increasing repeat length due to increased structural polymorphism and valency^55^, and LLPS can be initiated by GQs formed with the RNA^56^. LLPS is a biological phenomenon where biomacromolecules demix into two phases, a liquid within a liquid; this process is associated with membraneless organelle formation and can be driven by osmotic stress, dehydration, and/or specific molecular crowding microenvironments within the cell^38^. It has been previously shown that in the presence of dehydrating or osmotic stress conditions, such as with the molecular crowding agent PEG-200, DNA GQs frequently adopt different structural preferences than when under environmental conditions created by the molecular crowding agents Ficoll-70 or physiological crude intracellular extracts^40,41^. Consistent with these studies, our current results indicate that the C9-NRE GQ-AP topology is the preferred DNA GQ structure in molecular crowding conditions created by Ficoll that mimic the nucleoplasm. Whereas, in molecular crowding conditions created by PEG which may better mimic dehydrating or osmotic stress conditions of membraneless organelles or microenvironments of cellular LLPS, the GQ-P topology may be the preferred C9-NRE DNA GQ structure. Therefore, different DNA structures, repeat length, molecular crowding conditions, and LLPS are important factors to consider when modeling C9-NRE-linked disease mechanisms and for identifying therapies targeting specific DNA structures within certain cellular microenvironmental conditions.

The DNA structures formed within the C9-NRE mutation could provide repeat-length dependent therapeutic opportunities in patients. For example, the combination of large C9-NRE repeat track lengths and potential formation of persistent non-canonical nucleic acid structures can impede RNA/DNA polymerase processivity within the NRE locus^7,57^. Therefore, larger repeat expansions may provide greater pharmacological opportunities to stabilize these non-canonical nucleic acid structures and further impede transcription within the *C9-NRE* locus, which might result in the overall reduction of potential gain-of-function pathogenic mechanisms, such as ribonucleoprotein sequestration by repeat-containing RNA foci or the unconventional translation of repeat-containing transcripts leading to DPR proteinaceous toxicity^12–14^. Consistent with this idea, in C9-NRE disease models and patient tissue, key transcriptional elongation factors that increase RNA polymerase fidelity in the NRE region also increase RNA foci and DPR gain of-function toxicity^41–43^. Therefore, we posit that increasing the prevalence and/or stability of DNA GQ structures within the C9-NRE might decrease C9-NRE-linked gain-of-function pathogenic mechanisms.

The use of small-molecules targeting C9-NRE GQ-AP or GQ-P structures could provide opportunities to modulate the C9-NRE locus by altering the structural stabilities or protein-facilitated DNA GQ structural resolution. Antisense oligos or small molecules used in disease models have recently demonstrated that nucleic acids can be valuable upstream targets for treating downstream effects of neurological disorders and cancers^20–22^. Specifically, it was shown that small molecules that preferentially bind RNA GQs versus DNA GQs formed by the C9-NRE show efficacy in ameliorating C9-NRE-linked disease pathogenesis^19^, although the efficacy in ameliorating disease by targeting C9-NRE DNA GQs directly has not been addressed in this or other reported pharmacological studies. Small-molecules derived from diets, such as the polyphenols resveratrol or folate, have been shown to bind to GQ structures in vitro and result in altered global DNA methylation and/or GQ formation in cells^58^, which suggests that the combination of diet and persistent GQ formation could modify C9-NRE-linked disease pathogenesis. It is well-known that general DNA GQ-binding compounds often stack on guanine tetrads, which, in the case of C9-NRE GQs, can further modulate the motility of the cytosine-containing loop regions and therefore the accessibility of the cytosine residues to epigenetic modifiers such as TETs or the GQ-binding DNMTs^48^. Moreover, integration of the binding of cytosine-containing loop regions into the design of small molecules could reduce loop motility and protect them from further epigenetic modifications. Therefore, our identification of GQ topological preferences coupled with our molecular dynamic structural ensembles in this work could aid in identifying small molecules that bind C9-NRE DNA GQ structures with high efficacy to possibly treat debilitating diseases linked to this mutation. However, further work is required to fully appreciate the mechanistic relationship between C9-NRE DNA structural polymorphisms and the pathogenesis of C9-NRE-linked diseases, as well as to determine if modulating DNA GQ structures within the NRE can alter disease pathogenesis.

## Methods

### Formation of G-quadruplexes

For circular dichroism (CD) experiments, *C9*-22mer or methyl *C9*-22mer were used at a concentration of 10 µM in the presence of 10 mM Tris–HCl, pH 7.5, ± 100 mM KCl/LiCl and heated at 98 °C for 5 min, then cooled to room temperature at a rate of 1 °C per minute in a thermocycler.

### Circular dichroism

CD spectra for oligonucleotides were collected in 10 mM Tris–HCl, pH 7.5, ± 100 mM KCl/LiCl. Varying concentrations of PEG-200 (0, 10, 20, or 30% v/v) and/or Ficoll-70 (0, 10, 25, 50 mg/mL), were used to simulate varying cellular crowding conditions. In all in vitro experiments, DNA 22-mer and 48-mer oligos were used at a concentration of 10 µM and 5 µM concentrations, respectively. CD measurements were performed on a Jasco J-810 polarimeter using previously described parameters with minor modifications^7^. Specifically, parameters were set to the following: scan range 220–230 nm, scan speed 50 nm/min, 2 s response time, 1 nm bandwidth, and 3 acquisitions. CD spectra were obtained at 25 °C.

CD spectra melt curves were performed from a temperature range of 25 °C to 90 °C (the highest temperature accessible on this specific instrument) by monitoring absorbance at 295 nm for GQ-AP. Spectra were smoothed in Prism 7 using 4 neighbors on each size and 2nd order smoothing polynomial.

### CD spectral decomposition

CD spectra were decomposed assuming that each spectrum was a linear combination of the GQ-AP and GQ-P spectrum measured in the presence of 100 mM KCl and 100 mM KCl with 30% PEG, respectively, using the equation: $Spectrum=(AP)x+(P)y$. In this equation $AP$ is the GQ-AP component spectrum multiplied by the coefficient $x$, and $P$ is the GQ-P component spectrum multiplied by the coefficient $y$, with the constraints that $x+y=1$. The coefficients were calculated using the *fmincon* function in Matlab, minimizing the squared residual difference between the $(AP)x+(P)y$ and the measured spectra, $Spectrum$.

### Structural modeling of DNA G-quadruplexes

Anti-parallel DNA GQ (GQ-AP) was built from an antiparallel DNA GQ (PDB ID 5OPH) using the MD software YASARA version 18.4.24^59^. Briefly, the C8 bromine of the 8-bromodeoxyguanosine, residue 21, was modified to create the deoxyguanosine nucleotide base followed by energy minimization. To create the GQ-P, a parallel stranded DNA GQ was modified from the original file (PDB ID 139D). First, deoxythymidine residues 1 and 2 were deleted from all four individual oligonucleotides. Next the deoxythymidine residue 7 was mutated to deoxyguanosine. Then one deoxycytidine residue was added to the 3’ ends of each oligonucleotide. To form a continuous single oligonucleotide molecule, the 3’ hydroxyl group oxygen atoms of three deoxycytidine residue (of three strands) were bonded to the 5’ phosphate atom of the adjacent oligonucleotide. Energy minimization was performed to allow newly added bases to form loops, with G-tetrad bases being fixed to maintain position of G-tetrads. K^+^ ions were added to the planes between each of four stacks of tetrads for both GQ-AP and GQ-P.

For methylation molecular dynamic studies, the 5-methylcytosine nucleobase is not directly found in the YASARA library. Therefore, methylated GQs were created by adding a methyl group to the 5th atom of the 6 membered ring of all cytosine residues preceding a guanine residue. YASARA used “AutoSMILES” (http://www.yasara.org/autosmiles.htm) to generate new parameters for 5-methylcytosine using cytosine as the starting template followed by *charge assignment using AMBER’s AM1-BCC*^60^). Lastly atom types and parameters are assigned from GAFF (General Amber Force Field).

### MD simulations of DNA and RNA G-quadruplexes

MD simulations were performed within the YASARA graphical user interface using Amber14 with the force fields OL15^61^ + ff99bsc0. Simulations used an explicit solvent described by the TIP3P water model and a cubic periodic boundary that extended 20 Å around the GQ structure. Electrostatics were handled by the Particle mesh Ewald (PME) method with a cutoff of 8 Å for long range coulombic forces. The simulation cell was neutralized with 1.1% (w/v) K + at pH 7.4 with temperature maintained at 298 K (25 °C). The initial energy minimization and equilibration protocol begins in YASARA by predicting the ideal rotamers for the structure in the YASARA2 force field using implicit solvent and steepest-descent minimization. The hydrogen-bonding network is optimized and the structure is solvated in explicit solvent. Steepest descent minimization removes clashes followed by simulated annealing minimization where velocities of atoms are lowered by a factor of 0.9 per 10 steps to reach an energy minimum. The MD Simulation initiates when the simulated annealing energy of the system improves by less than 0.05 kJ/mol/atom over the duration of 200 steps. Simulations were then run for a duration of 1 µs with a time step of 2.5 fs. Atomic coordinates were recorded every 100 ps. MD trajectories were analyzed using YASARA macros, as well as standard self-created macros. Models were visualized and generated using Pymol. It is important to note the OL15 force field has been used successfully applied to MD simulations for DNA GQs^62–64^, however, the conformation and the stability calculated for non-canonical nucleic acid structures are force-field dependent, especially over relatively long MD simulation time courses^65,66^.

### Statistical analyses

All data was plotted using Graphpad Prism as the mean ± SEM unless otherwise noted. All *p*-values were calculated using an unpaired, two-tailed t-test.

## Supplementary Information

Supplementary Information.

## Data Availability

All PDB files used to generate the initial structures were obtained from the RCSB Protein Data Bank, https://www.rcsb.org, using the specific PDB files 5OPH and 139D.
