# Supplementary material for: The effects of molecular crowding and CpG hypermethylation on DNA G-quadruplexes formed by the C9orf72 nucleotide repeat expansion
Source: Sci Rep. 2021 Dec 1;11:23213. doi: 10.1038/s41598-021-02041-4 (PMC8636472; doi:10.1038/s41598-021-02041-4)
Supplement: Supplementary file 1 — Supplementary Information. [file 41598_2021_2041_MOESM1_ESM.docx]

**SUPPLEMENTARY INFORMATION**

| **Oligo Name** | **Sequence** | **Experiment** | **Application** |
| --- | --- | --- | --- |
| *C9*-22mer | GGGGCCGGGGCCGGGGCCGGGG | CD | Anti-parallel and parallel G-quadruplexes |
| m*C9*-22mer | GGGGCmCGGGGCmCGGGGCmCGGGG | CD | CpG methylated anti-parallel and parallel G-quadruplexes |
| *C9*-48mer | GGGGCCGGGGCCGGGGCCGGGGCC GGGGCCGGGGCCGGGGCCGGGGCC | CD | Anti-parallel and parallel G-quadruplexes |
| m*C9*-48mer | GGGGCmCGGGGCmCGGGGCmCGGGGCmCGGGGCmCGGGGCmCGGGGCmCGGGGCC | CD | CpG methylated Anti-parallel and parallel G-quadruplexes |

### **Supplementary Table S1 |** List of oligonucleotides used in these experiments. All oligos, listed 5′ to 3′, were purchased from IDT.


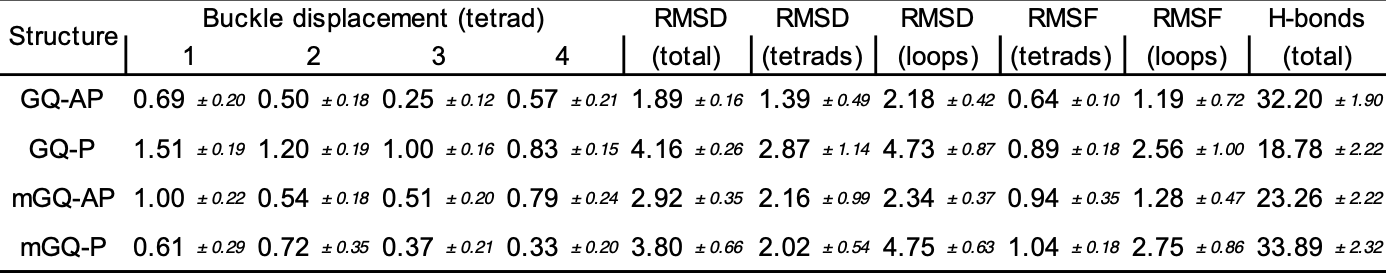


### **Supplementary Table S2 |** Summary of key results from molecular dynamic simulation performed over 1 µs on non-methylated (GQ) and methylated (mGQ) C9-NRE GQs. Data is shown as Mean ± STD.

### ****

### **Supplementary Figure S1 | Longer C9orf72 NRE repeat lengths show similar CD spectra profiles in response to specific molecular crowding environments as observed with smaller repeat lengths**. (**A**) CD spectra obtained for the (G_4_C_2_)_8_, *C9*-48mer, at 5 uM demonstrates that longer repeat length forms GQ-AP topology in the presence of 100 mM KCl but not 100 mM LICl. (**B**) CD spectra profiles for the *C9*-48mer show that there is an increasing GQ-AP to GQ-P topological transition with increasing [PEG-200] in 100 mM KCl. (**C**) Increasing the molecular crowding agent [Ficoll-70] has minimal effects on the *C9*-48mer CD spectra profiles. (**D**) Varying combinations of the molecular crowding agents PEG-200 and Ficoll-70 show that CD spectra topological profiles for the *C9*-48mer are largely influenced by [PEG-200].

### **Supplementary Figure S2** | **CD spectra can be recreated using spectral components to determine GQ topological distributions in the presence of different molecular crowding agents**. (**A**) The recreated spectra that were generated by fitting each individual CD spectra with a linear combination of spectral components for GQ-AP spectra (0% [PEG-200] spectra) and GQ-P (30% [PEG-200] spectra) for the *C9-*22mer in 100 mM KCl shown in Figure 1C. The calculated residuals for the spectral data versus the fit are shown immediately below the fit spectra. Increasing [PEG-200] (are represented by the black to red line transition). The calculated fractional component contribution of GQ-AP and GQ-P (y-axis) relative to increasing [PEG-200] for the *C9-*22mer is shown at the bottom. (**B**) The calculated fractional components contribution of GQ-AP and GQ-P, which were calculated as in **A**, for increasing [Ficoll-70]. (**C**) The effects of the different combinations of varying Ficoll-70 and PEG-200 concentrations on the distribution of GQ-AP and GQ-P as calculated in **A**.

### ****

### **Supplementary Figure S3 | CD spectra can be recreated to calculate the GQ topological distributions for the mC9-22mer in response to different molecular crowding conditions.** (**A**) The recreated spectra were generated by fitting each individual CD spectra with a linear combination of spectral components for GQ-AP spectra (0% [PEG-200] spectra) and GQ-P (30% [PEG-200]) spectra) for 10 µM of the *C9-*22mer in 100 mM KCl. The calculated residuals for the spectral data versus the fit are shown immediately below the fit spectra. Increasing [PEG-200] are represented by the black to red line transition. The calculated fractional component contribution of GQ-AP and GQ-P (y-axis) relative to increasing [PEG] for the *mC9-*22mer is shown at the bottom. (**B**) Increasing [Ficoll-70] shows negligible effects on the CD spectra profiles and thus the GQ topological distribution for the *mC9*-22mer. (**C**) The combination of varying PEG and/or Ficoll-70 concentrations show that the molecular crowding agent PEG largely determines the CD spectra profile and GQ topological distributions for the *mC9*-22mer.

### ****

### **Supplementary Figure S4 | 5mC hypermethylation on longer C9-NRE DNA repeats shows similar GQ profiles as both nonmethylated longer and shorter repeats in the presence and absence of different molecular crowding agents**. The representative CD spectra shows typical profiles for GQ-AP in the presence of KCl (solid black line) but not in the presence LiCl (dotted black line) for the m*C9*-48mer at 5 µM concentration. The CD spectrum in the presence of Ficoll-70 shows that the m*C9*-48mer forms GQ-AP structures (green line) while in the presence of PEG-200 (red line) it forms GQ-P structures.


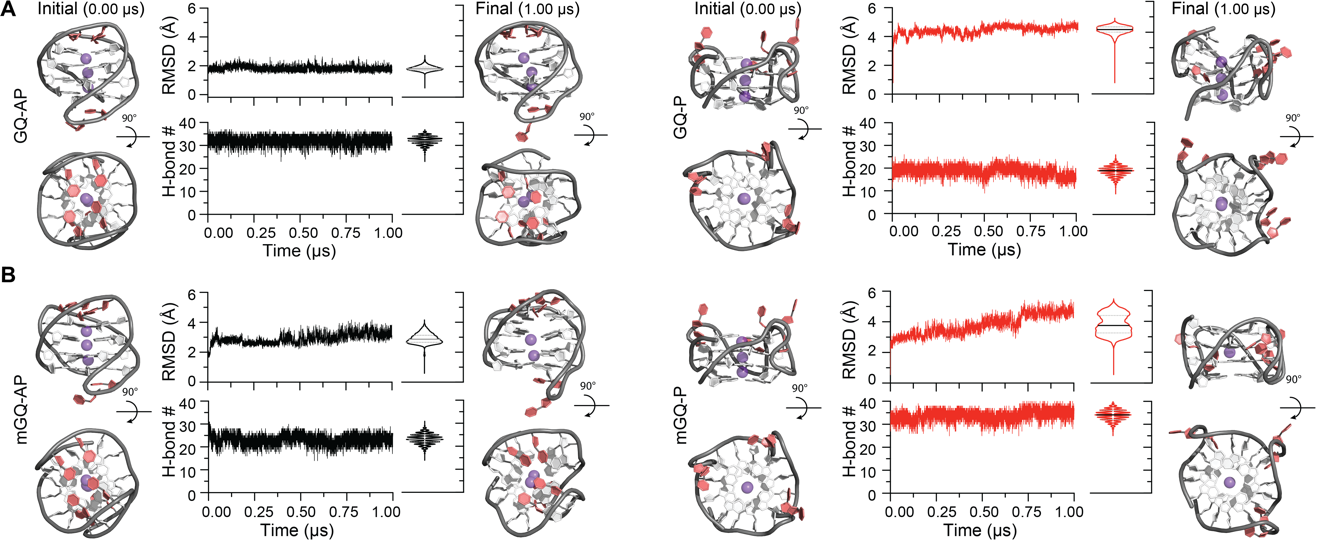


### **Supplementary Figure S5 | Nonmethylated and methylated C9-NRE DNA GQ structures show minimal overall structural variation in MD simulations**. (**A**) RMSD and hydrogen bond numbers are plotted to evaluate the structural stability of GQ-AP (black line) or GQ-P (red line) over a 1 µs MD simulation. Violin plots are for the integrated values over the entire simulation, with solid lines depict the mean and dotted lines showing the 25^th^ and 75^th^ quartiles. Initial structures with 90° rotations and final structure with 90° rotations are depicted on the left and right of the RMSD graphs, respectively. (**B**) 5mC methylation of the GQ structures shows similar stability and dynamics as the nonmethylated structural counterparts in **A**.

###

### **Supplementary Figure S6 | CpG methylation of the C9-NRE has minimal effects on the structural dynamics of GQs in MD simulations.** The plotted bar graphs show RMSD (left) and RMSF (right) comparisons between nonmethylated (GQ-AP or GQ-P) and methylated (mGQ-AP or mGQ-P) GQ structures calculated from the MD simulations performed over 1 µs. Each G residue is considered part of a tetrad and is shown as a square. All C residues are considered loop residues and are shown as a circle. Clear or gray filled bars designate tetrads or loops, respectively. Data are plotted as the mean ± SEM. All *p-values* were calculated using a standard unpaired T-test with the *p-values* = not significant, ≤ 0.01, ≤ 0.001, or ≤0.001 denoted as: ns, **, ***, or ****, respectively.
